# Carbohydrate Syntrophy enhances the establishment of *Bifidobacterium breve* UCC2003 in the neonatal gut

**DOI:** 10.1038/s41598-018-29034-0

**Published:** 2018-07-13

**Authors:** Mary O’Connell Motherway, Frances O’Brien, Tara O’Driscoll, Patrick G. Casey, Fergus Shanahan, Douwe van Sinderen

**Affiliations:** 1 APC Microbiome Ireland, National University of Ireland, Cork, Western Road, Cork, Ireland; 2 School of Microbiology, National University of Ireland, Cork, Western Road, Cork, Ireland; 3 Department of Medicine, National University of Ireland, Cork, Western Road, Cork, Ireland

## Abstract

The non-digestible oligosaccharide fraction of maternal milk represents an important of carbohydrate and energy source for saccharolytic bifidobacteria in the gastrointestinal tract during early life. However, not all neonatal bifidobacteria isolates can directly metabolise the complex sialylated, fucosylated, sulphated and/or N-acetylglucosamine-containing oligosaccharide structures present in mothers milk. For some bifidobacterial strains, efficient carbohydrate syntrophy or crossfeeding is key to their establishment in the gut. In this study, we have adopted advanced functional genomic approaches to create single and double in-frame deletions of the N-acetyl glucosamine 6-phosphate deacetylase encoding genes, *nagA1* and *nagA2*, of *B*. *breve* UCC2003. *In vitro* phenotypic analysis followed by *in vivo* studies on co-colonisation, mother to infant transmission, and evaluation of the relative co-establishment of *B*. *bifidum* and *B*. *breve* UCC2003 or UCC2003ΔnagA1ΔnagA2 in dam-reared neonatal mice demonstrates the importance of crossfeeding on sialic acid, fucose and N-acetylglucosamine-containing oligosaccharides for the establishment of *B*. *breve* UCC2003 in the neonatal gut. Furthermore, transcriptomic analysis of *in vivo* gene expression shows upregulation of genes associated with the utilisation of lactose, sialic acid, GlcNAc-6-S and fucose in *B*. *breve* UCC2003, while for UCC2003ΔnagA1ΔnagA2 only genes for lactose metabolism were upregulated.

## Introduction

Bifidobacteria are among the earliest and most abundant bacterial colonisers of the neonatal gut where their presence is associated with a myriad of benefits to the host intestinal, metabolic and immune health^1^. While the genus *Bifidobacterium* comprises more than 50 species/subspecies, the dominant infant associated species include *Bifidobacterium longum* subsp. *longum*, *Bifidobacterium longum* subsp *infantis*, *Bifidobacterium bifidum*, *Bifidobacterium breve*, *Bifidobacterium pseudocatenulatum*, *Bifidobacterium*. *catenulatum*, *Bifidobacterium kashiwanohense*, and *Bifidobacterium adolescentis*^2,3^. The dominance of bifidobacteria in breastfed infants has been attributed to their ability to utilise human milk oligosaccharides (HMOs). In particular, strains of *B*. *longum* subsp. *infantis* and *B*. *bifidum* have been studied extensively for their ability to utilise host derived carbohydrates and have been found to harbour dedicated, yet distinct, metabolic capabilities for the utilisation of HMOs^4,5^ while more recently specific strains of *B*. *longum* subsp. *longum*, *B*. *pseudocatenulatum* and *B*. *kashiwanohense* have been investigated for their ability to utilise fucosyllactose, the dominant oligosaccharide in human milk^6,7^. Intriguingly, the ability to utilise HMOs would seem to be a variable trait among infant-derived strains of *B*. *breve*, with some strains exhibiting good growth on purified HMOs under *in vitro* conditions, while other *B*. *breve* strains exhibit no appreciable growth^2^. The presence and isolation of *B*. *breve* strains that do not directly metabolise HMOs would suggest that these strains likely adopt carbohydrate syntrophy to allow their establishment in the infant gut. We hypothesise that *B*. *breve*, and perhaps other *Bifidobacterium* sp, can exploit the extracellular glycosyl-hydrolase activity of other (bifido)bacterial members of the infant gut microbiome as a source of fermentable carbohydrates to support growth in the intestine.

We have previously shown that *B*. *breve* UCC2003, a nursling infant stool isolate, can efficiently utilise sialic acid, but not host derived 3′ sialyllactose, as sole carbohydrate source^8^. However, *B*. *breve* UCC2003 can crossfeed on released sialic acid derived from the extracellular metabolism of 3′ sialyllactose by *B*. *bifidum* PRL2010 during an *in vitro* sequential co-culture experiment^8^. Similarly *B*. *breve* UCC2003 cannot directly utilise the most abundant HMO in mother’s milk, fucosyllactose (2′FL or 3′FL), but can crossfeed on monosaccharides, including fucose, that are released during co-culture with *B*. *bifidum* PRL2010 in mMRS medium supplemented with porcine mucin^9^.

N-acetylglucosamine deacetylase (NagA) activity is central to the metabolism of HMOs and host derived carbohydrates, whereby NagA catalyses the conversion of N-acetylglucosamine 6-phosphate to glucosamine 6-phosphate. The genome of *B*. *breve* UCC2003, ate, harbours 2 genes, namely Bbr_0846 and Bbr_1247 (designated *nag*A1 and *nag*A2, respectively) whose protein products, NagA1 and Nag A2, respectively, share 74% identity and are predicted to encode N-acetyl glucosamine deacetylase activity. Expression of *nag*A1is significantly upregulated during growth of *B*. *breve* UCC2003 in mMRS medium supplemented with the host derived sulphated carbohydrate N-acetyl glucosamine-6-sulphate (GlcNAc-6-S) or Lactosamine-HCl, while expression of *nag*A2 is significantly upregulated during growth of *B*. *breve* UCC2003 in mMRS medium supplemented with sialic acid, LNT, LNnT, GlcNAc-6-S and also when *B*. *breve* UCC2003 is grown in co-culture with *B*. *bifidum* PRL2010 in medium supplemented with mucin^8–11^. Previously a *B*. *breve* UCC2003-nagA2 insertion mutant strain was found to exhibit growth comparable to that of the parent strain, *B*. *breve* UCC2003, in medium supplemented with sialic acid as sole carbohydrate source suggesting that NagA1 may compensate in the absence of NagA2 activity^8^.

To extend our understanding of bifidobacterial mutualism and carbohydrate syntrophy in the gut we adopted advanced functional genomics to create single- and double-deletion isogenic strains of the NagA-encoding genes of *B*. *breve* UCC2003. The resulting strains were examined, as compared to the parent strain, for their ability to metabolise particular host-derived carbohydrates. In addition, the *B*. *breve* strains were examined for their cross-feeding capability and ability to establish, in the presence of *B*. *bifidum*, in the gut of dam fed neonatal mice.

## Results

### Phenotypic analysis of *B*. *breve* UCC2003 strains harbouring deletions of the *N*-acetyl glucosamine deacetylase encoding genes, *nag*A1 and *nag*A2

**Figure 1:**
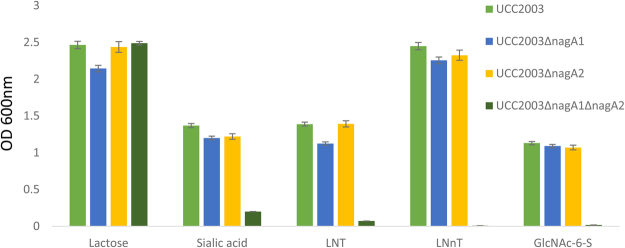
Final optical densities (OD 600 nm) of *B*. *breve* UCC2003, UCC2003ΔnagA1, UCC2003ΔnagA2 or UCC2003ΔnagA1ΔnagA2 following 24 hours growth in mMRS medium supplemented with lactose, sialic acid, LNT, LNnT or GlcNAc-6-S at 0.5% w/v final concentration.

To establish if N-acetyl glucosamine deacetylase activity is essential for the metabolism of sialic acid and other host derived carbohydrates isogenic *B*. *breve* UCC2003 derivative strains harbouring inframe deletions of *nag*A1 or *nag*A2, and a double (*nag*A1*nag*A2) deletion strain were created, and designated *B*. *breve* UCC2003ΔnagA1, UCC2003ΔnagA2 or UCC2003ΔnagA1ΔnagA2, respectively. These three mutants were compared to *B*. *breve* UCC2003 for their ability to utilise lactose, sialic acid, Lacto-*N*-tetraose (LNT), Lacto-*N*-neotetraose (LNnT), or N-Acetyl-D-glucosamine-6-O-sulfate (GlcNAc-6-S) as the sole carbohydrate source. All strains achieved final optical densities (OD 600_nm_) greater than 2.0 in mMRS medium supplemented with lactose (positive control). In mMRS medium supplemented with sialic acid, LNT, LNnT or GlcNAc-6-S *B*. *breve* UCC2003, UCC2003ΔnagA1, UCC2003ΔnagA2 achieved comparable final optical density values for each carbohydrate substrate, while growth of *B*. *breve* UCC2003ΔnagA1ΔnagA2 was impaired (Fig. 1).

### Carbohydrate syntrophy enhances the establishment of *Bifidobacterium breve* UCC2003 in the neonatal gut

**Figure 2:**
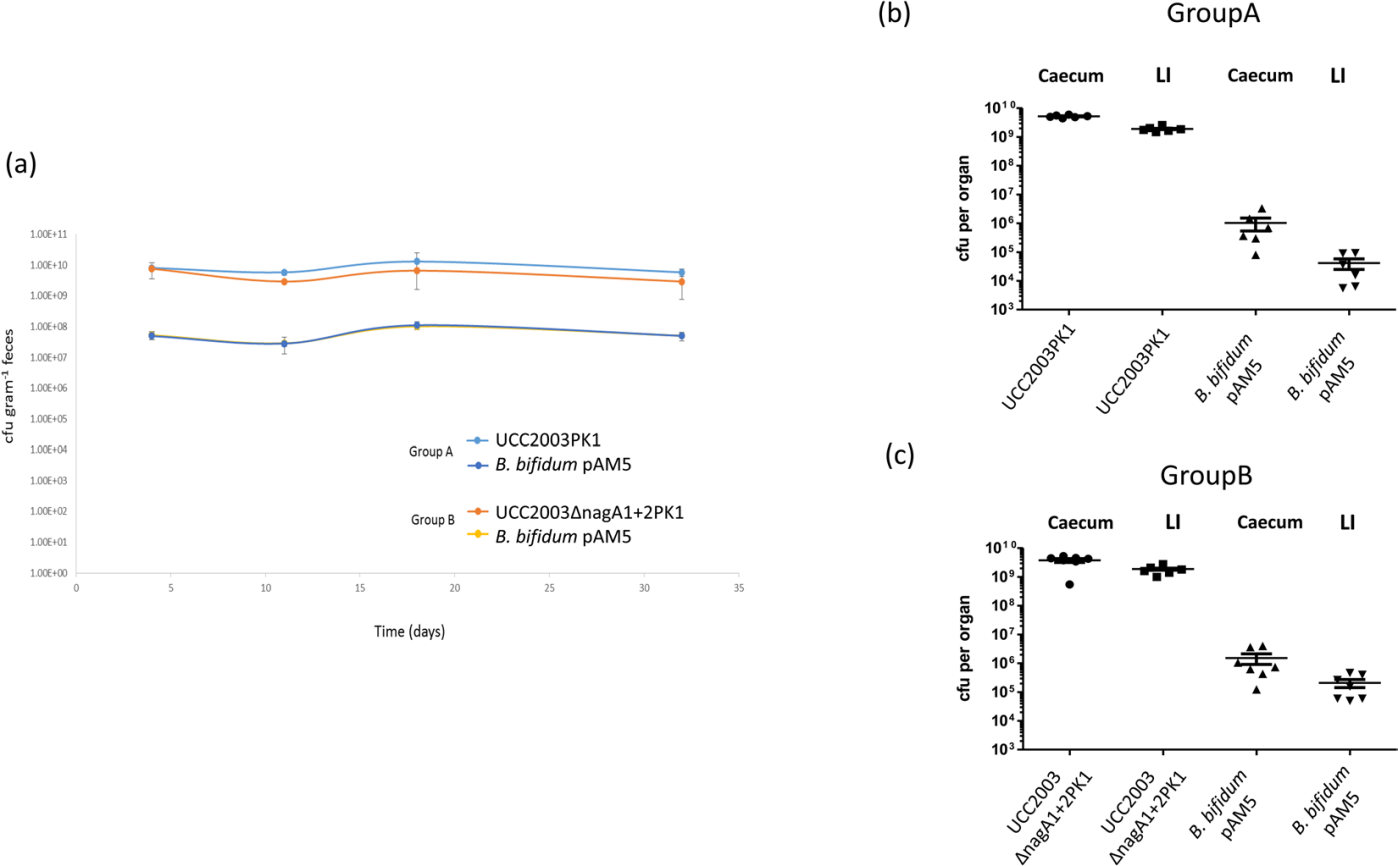
Co-colonisation of *B*. *breve* UCC2003PK1 and *B*. *bifidum* PAM5 or *B*. *breve* UCC2003ΔnagA1 + 2′ PK1 and *B*. *bifidum* PAM5 in pregnant germ free C57BL/6 mice (**a**) Recovery of *B*. *breve* UCC2003PK1 (pale blue) and *B*. *bifidum* PAM5 (dark blue) or *B*. *breve* UCC2003ΔnagA1ΔnagA2 (orange) and *B*. *bifidum* PAM5 (yellow) from murine fecal samples of C57BL/6 co-associated mice over 4 week trial period. (**b**) Comparison of numbers of *B*. *breve* UCC2003PK1 and *B*. *bifidum* PAM5 or (**c**) *B*. *breve* UCC2003ΔnagA + 2PK1 and *B*. *bifidum* PAM5 and recovered from the caecum and the large intestine of co–associated animals.

**Figure 3:**
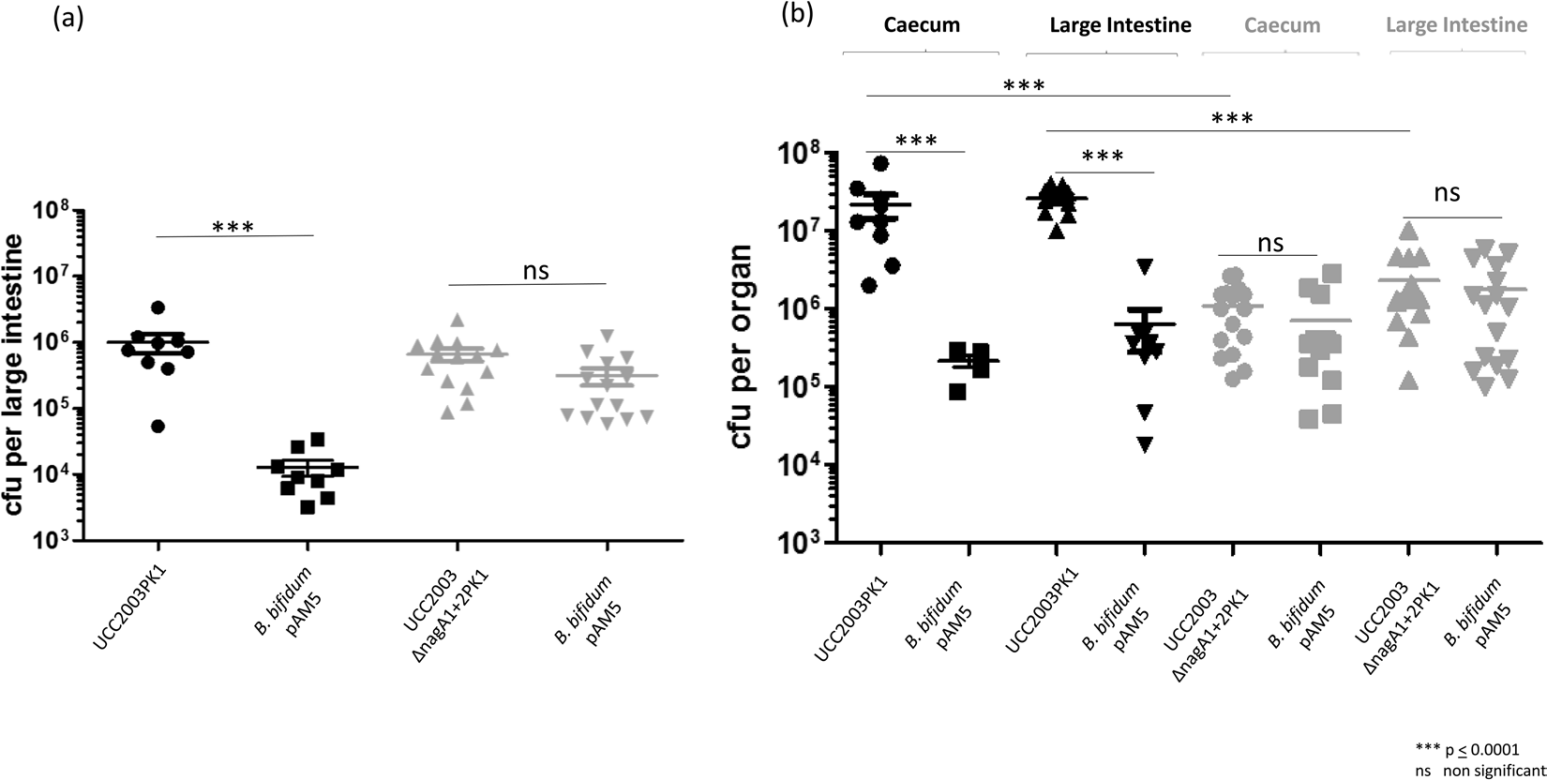
Enumeration of Bifidobacteria from the intestine of dam reared mice. Enumeration of *B*. *breve* UCC2003PK1 and *B*. *bifidum* PAM5 (shaded black), or *B*. *breve* UCC2003ΔnagA1 + 2PK1 and *B*. *bifidum* PAM5 (shaded grey) on cull day 1 (**a**) or 2 (**b**) from the intestine or caecum of dam reared neonatal mice.

In order to establish if the ability to metabolise host derived carbohydrates enhances the numbers of *B*. *breve* UCC2003 in the gastrointestinal tract of Dam-reared neonatal murine pups, a co-association study was performed. Groups of 7 pregnant C57BL/6 Germ free mice were administered a single dose of 1 × 10^9^ cfu of each *B*. *bifidum* PAM5 and *B*. *breve* UCC2003PK1, or *B*. *bifidum* PAM5 and UCC2003∆nagA1 + 2PK1. Fecal samples were collected weekly during the trial period to enumerate bifidobacterial shedding and determine the relative colonisation ability of *B*. *bifidum* PAM5, *B*. *breve* UCC2003PK1 or *B*. *breve* UCC2003∆nagA1 + 2PK1. Interestingly, despite administration at equal levels, *B*. *breve* UCC2003 or *B*. *breve* UCC2003∆nagA1 + 2PK1 colonised the pregnant mice at approximately 100 fold higher level as compared to *B*. *bifidum* PAM5 (Fig. 2a). This difference in colonisation ability between *B*. *breve* UCC2003 derivatives and *B*. *bifidum* PAM5 was also reflected in the numbers of each strain recovered from the caecum and large intestine of the adult mice at the end of the trial period (Fig. 2b and c). The first litters of pups were born 9 days after administration of the *Bifidobacterium* strains, with all pups born within a period of 5 days. For Group A, administered *B*. *bifidum* PAM5 and *B*. *breve* UCC2003PK1, just three of the seven mothers produced litters of pups, while for Group B administered *B*. *bifidum* PAM5 and *B*. *breve* UCC2003∆nagA1 + 2PK1 five of the seven mothers gave birth to litters of pups (Table 1). All pups were allowed to feed from their mothers and at 2 time points postpartum, and while the pups were exclusively dam reared, half of each group was culled for enumeration of each *Bifidobacterium* strain in the caecum or large intestine based by plate counting with selection based on antibiotic resistance. For Group A, where pregnant mothers were administered *B*. *bifidum* PAM5 and *B*. *breve* UCC2003PK1, mother-to-pup transmission of both *Bifidobacterium* strains was observed with *B*. *breve* UCC2003PK1 present at a level that was almost 100-fold higher as compared to that achieved by *B*. *bifidum* PAM5 in the large intestine of the pups at each time point (p ≤ 0.001on cull day 1 and 2), achieving average levels of 1 × 10^6^ cfu and 1.3 × 10^4^ cfu, respectively, on cull day 1, while levels of 2.59 × 10^7^ cfu and 6.3 × 10^5^ cfu, respectively were recovered from the large intestine on cull day 2 (Fig. 3a and b). Similarly, for Group B, where mothers were administered *B*. *bifidum* PAM5 and *B*. *breve* UCC2003∆nagA1 + 2PK1, mother-to-pup transmission of both bifidobacterial strains was observed. However, the numbers of *B*. *breve* UCC2003∆nagA1 + 2PK1 and *B*. *bifidum* PAM5 were not significantly different on either cull day. Average levels of *B*. *breve* UCC2003∆nagA1 + 2PK1 and *B*. *bifidum* PAM5 recovered from the large intestines were 6.5 × 10^5^ and 3.1 × 10^5^ respectively, on the first cull day, with levels of each strain increasing to 2.3 × 10^6^ cfu and 1.77 × 10^6^ cfu, respectively on cull day 2 (Fig. 3a and b).

**Table 1:** Number of pups born to each C57Bl/6 mother and number of dam-reared pups culled at each timepoint.

	Mother/litter	Number of pups in litter	Cull day: 1	Cull day: 2
			Number of pups (age in days)	Number of pups (age in days)
Group A: administered *B. bifidum* PAM5 and UCC2003PK1	A1	8	4 (10 days)	4 (14 days)
	A2	0		
	A3	3	2 (11 days)	1 (15 days)
	A4	0		
	A5	0		
	A6	0		
	A7	7	3 (11 days)	4 (15 days)
			**Total 9 animals**	**Total 9 animals**
Group B: administered *B. bifidum* PAM5 and UCC2003∆nagA1 + 2PK1	B1	5	2 (8 days)	3 (12 days)
	B2	6	3 (12 days)	3 (16 days)
	B3	8	4 (10 days)	4 (14 days)
	B4	0		
	B5	7	3 (10 days)	4 (14 days)
	B6	4	2 (9 days)	2 (13 days)
	B7	0		
			Total 14 animals	Total 16 animals

### *B*. *breve* UCC2003 or UCC2003∆nagA1 + 2 transcriptome during colonisation of dam-reared neonatal mice

To determine the *B*. *breve* UCC2003 or UCC2003∆nagA1 + 2 genes that are differentially transcribed in the gut of the dam-reared neonatal mice relative to the transcriptional profile under laboratory conditions, total bacterial RNA was isolated from the large intestines of neonatal mice harbouring *B*. *breve* UCC2003PK1 and *B*. *bifidum* PAM5 or *B*. *breve* UCC2003∆nagA1 + 2PK1 and *B*. *bifidum* PAM5. The RNA was reverse transcribed and the cDNA used to determine the *in vivo* transcriptome of each strain as compared to an exponential phase culture *B*. *breve* UCC2003 grown in mMRS supplemented with ribose. A total of 74 *B*. *breve* UCC2003 genes were significantly upregulated *in vivo* (≥3 fold; p < 0.001) and 95 downregulated (≥8 fold; p < 0.001), while for *B*. *breve* UCC2003∆nagA1 + 2PK1 23 genes were significantly upregulated *in vivo* (≥3 fold; p < 0.01) and 88 downregulated (≥8 fold; p < 0.001) relative to the control. The most highly upregulated genes were in loci dedicated to carbohydrate metabolism. In particular genes Bbr_1551 and Bbr_1552, dedicated to lactose transport and metabolism were significantly upregulated in both *B*. *breve* UCC2003PK1 and *B*. *breve* UCC2003∆nagA1 + 2PK1 under *in vivo* conditions, while genes in the sialic acid metabolism locus (Bbr_0160-Bbr_0172), the fucose metabolism locus (Bbr_1741–1745), and the nag and sulphatase locus (Bbr_0846- Bbr_0853) were significantly upregulated in *B*. *breve* UCC2003PK1 but not in *B*. *breve* UCC2003∆nagA1 + 2PK1 under *in vivo* conditions (Table 2). In addition, genes predicted to be remnants of an *N*-acetylglucosamine PTS system (Bbr_1878-Bbr_1880) were also upregulated in *B*. *breve* UCC2003PK1. Expression of the bile salt hydrolase encoding gene (BBr_1520) and several genes encoding hypothetical membrane proteins were upregulated in both *B*. *breve* UCC2003PK1 and *B*. *breve* UCC2003∆nagA1 + 2PK1under *in vivo* conditions indicating that *B*. *breve* UCC2003 expresses specific sets of genes in response to the *in vivo* environment and during feeding on mother’s milk (Table S1).

**Table 2:** Differential expression of carbohydrate utilisation gene loci in *B*. *breve* UCC2003PK1 or *B*. *breve* UCC2003ΔnagA1ΔnagA2PK1 under *in vivo* conditions in dam-reared neonatal mice.

Locus tag	Function	Fold upregulation UCC2003	Fold upregulation UCC2003ΔnagA1ΔnagA2
**Sialic acid metabolism**			
Bbr_0160	Conserved hypothetical protein	2.80^a^	—^b^
Bbr_0161	Conserved hypothetical protein in ROK family	—	—
Bbr_0162	N-acetylmannosamine-6-phosphate 2-epimerase	4.52	2.39
Bbr_0163	Hydrolase	—	—
Bbr_0164	Substrate binding protein	11.89	—
Bbr_0165	ABC transport system permease protein	5.36	—
Bbr_0166	ABC transport system ATP-binding protein	7.60	3.11
Bbr_0167	ABC transport system ATP-binding protein	7.57	—
Bbr_0168	*nanA* N-acetylneuraminate lyase	20.35	4.7
Bbr_0169	Glucosamine-6-phosphate isomerase	9.00	—
Bbr_0171	Sialidase A	4.49	—
Bbr_0172	ATPase	—	—
***nag*** **genes and metabolism of sulphated sugars**			
Bbr_0846	*nagA1* N-acetylglucosamine-6-phosphate deacetylase	2.0	—
Bbr_0847	*nagB2* Glucosamine-6-phosphate isomerase	3.78	—
Bbr_0851	Glucose/fructose transport protein	5.55	2.95
Bbr_0852	Sulfatase family protein	2.04	—
Bbr_0853	*atsB* Arylsulfatase regulator (Fe-S oxidoreductase)	—	—
Bbr_0854	Conserved hypothetical	2.12	—
Bbr_0855	Hypothetical protein	5.14	—
**Lactose metabolism**			
Bbr_1550	Hypothetical protein	6.24	4.94
Bbr_1551	*lacS* Galactoside symporter	4.50	3.86
Bbr_1552	*lacZ6* Beta-galactosidase	4.41	4.25
**Fucose metabolism**			
Bbr_1741	Conserved hypothetical protein	4.91	—
Bbr_1742	L-fucose permease	3.16	—
Bbr_1743	Short chain dehydrogenase	3.35	—
Bbr_1744	Mandelate racemase	3.33	—
Bbr_1745	Transcriptional regulator	—	—
**Remnants of Putative N-acetylglucosamine PTS system**			
Bbr_1878	Hypothetical protein	3.27	—
Bbr_1879	PTS system, glucose-specific IIABC component	6.15	—
Bbr_1880	PTS system, N-acetylglucosamine-specific IIBC component	6.77	3.78

^a^Fold upregulation ≥2 and p value ≤ 0.001. ^b^Values below threshold.

## Discussion

The predominant carbohydrate in human milk is lactose which is found at a concentrations ranging from of 60–70 g L^−1^. The concentration of HMOs, with a degree of polymerisation ≥3, in human milk varies considerably, with values of 22–24 g L^−1^ in colostrum, and 12–13 g L^−1^ in mature milk^12^. HMOs comprise more than 200 oligosaccharide structures that are classified as Type I or Type II oligosaccharides based the disaccharide lacto-*N*-biose or *N*-acetyllactosamine, respectively, at their reducing end. Fucosyllactose, Lacto-*N*-tetraose and Lacto-*N*-fucopentaose are the most abundant HMOs, and these 3 oligosaccharides can comprise up to 55% of the HMO content of human milk^12–14^. The predominance of Type I oligosaccharides is a feature of human milk, also found in mother’s milk of chimpanzees and elephants, however, among other mammals and marsupials Type II oligosaccharides tend to dominate^15^.

In this study we sought to establish the role of the *B*. *breve* N-acetylglucosamine deacetylases, NagA1 and NagA2, in utilising host-derived carbohydrates including HMOs. In contrast to the parental strain, *B*. *breve* UCC2003, and the single deletion strains, *B*. *breve* UCC2003∆nagA1or UCC2003∆nagA2, *B*. *breve* UCC2003∆nagA1∆nagA2 was incapable of utilising sialic acid, LNT, LNnT or GlcNAc-6-S as sole carbohydrate source while this double *nag*A deletion strain exhibits growth comparable to the parent strain, and the single deletion strains, in medium supplemented with lactose.

We next wished to establish if the ability to metabolise host-derived oligosaccharides confers *B*. *breve* with a growth advantage in the neonatal gut. There has been a recent surge in publications describing the oligosaccharide composition of mother’s milk from various animal sources, however despite laboratory mice being used extensively for research purposes, deciphering the oligosaccharide fraction of murine milk has received relatively little attention. This is likely due to the difficulty in obtaining sufficient volumes of milk for analysis. Despite this obstacle, Prieto *et al*.^16^ successfully determined the oligosaccharide composition of murine milk and established that in addition to lactose, murine milk contains oligosaccharides including sialyllactose (3′SL and 6′SL) and fucosyllactose. Since *B*. *breve* UCC2003 does not directly utilise these milk oligosaccharides we adopted a co-inoculation strategy whereby two groups of 7 pregnant germ free mice were gnotobiotically co-colonised with *B*. *breve* UCC2003PK1 and *B*. *bifidum* PAM5 or *B*. *breve* UCC2003∆nagA1 + 2PK1 and *B*. *bifidum* PAM5. Interestingly, the *B*. *breve* strains colonised the pregnant adult mice at an approximately 2 log higher level as compared to *B*. *bifidum* PAM5. This is likely due to the ability of UCC2003 PK1 or UCC2003∆nagA1 + 2PK1 to metabolise starch, a major component of the adult rodent diet^17^. The higher numbers of either *B*. *breve* strain as compared to *B*. *bifidum* PAM5 were also reflected in the bacterial numbers in the caecum and large intestine of the adult mice at the end of the trial period. Despite being housed in the same animal room and under the same conditions just 3 of the 7 mothers in group A produced viable litters, while 5 of the 7 mothers in group B produced viable litters. We attribute this relatively low number of mothers producing litters to the C57Bl/6 mice being first time mothers, and had we used established C57Bl/6 breeding mice more viable litters may have been obtained. Despite this, we obtained sufficient pups to have two timepoints for analysis while the pups were exclusively dam-reared. For each group of mice we observed mother-to-pup transmission of the *Bifidobacterium* strains. Enumeration of *B*. *breve* UCC2003PK1 and *B*. *bifidum* PAM5 from the caecum or large intestine of the pups on either cull day showed an almost 2 log higher level of *B*. *breve* UCC2003PK1 as compared to *B*. *bifidum* PAM5, while the numbers of *B*. *breve* UCC2003∆nagA1 + 2PK1 or *B*. *bifidum* PAM5 were not significantly different.

This data clearly indicates that the ability to cross-feed on host-derived carbohydrates provides *B*. *breve* UCC2003 with a competitive advantage, as compared to it’s isogenic double mutant or *B*. *bifidum* PAM5, that allows this strain to establish to a higher level in the gut of the dam-reared neonatal mice. To validate our findings we determined the *in vivo* transcriptome of *B*. *breve* UCC2003PK1 or UCC2003∆nagA1 + 2 as compared to UCC2003 grown under *in vitro* conditions. The *in vivo* transcriptome of both strains shows significant upregulation of genes involved in lactose metabolism demonstrating that both *B*. *breve* UCC2003PK1 or *B*. *breve* UCC2003∆nagA1 + 2PK1 are utilising lactose as a carbohydrate source in the neonatal gut. However, gene loci involved in metabolism of sialic acid, fucose and sulphated sugars were significantly upregulated in the *in vivo* transcriptome of *B*. *breve* UCC2003, yet not in that of *B*. *breve* UCC2003∆nagA1 + 2PK1, demonstrating that sialic acid, fucose and sulphated sugars are a valuable carbohydrate source, and metabolised by *B*. *breve* in the neonatal gut. Interestingly, transcription of the bile salt hydrolase-encoding gene was upregulated in both strains under these *in vivo* conditions, as well as a number of genes encoding uncharacterised membrane spanning hypothetical protein, suggesting that they may mediate microbe-microbe, or microbe-host dialogue.

Collectively these data demonstrate that bifidobacterial mutualism and carbohydrate syntrophy occurs in the neonatal gut, thereby allowing *B*. *breve* strains that to not directly metabolise dominant host-derived oligosaccharides to efficiently cross-feed and achieve high numbers. In addition, these data suggest that the intracellular levels amino sugars *N*-acetylglucosamine and *N*-acetylgalactosamine or glucosamine 6-phosphate may be key intermediates regulating the expression of *B*. *breve* loci dedicated to metabolising host-derived oligosaccharides.

## Methods

### Bacterial strains, plasmids and culture conditions

Bacterial strains and plasmids used in this study are listed in Table 3. Bifidobacterial strains were routinely cultured in reinforced clostridial medium (RCM; Oxoid Ltd, Basingstoke, Hampshire, United Kingdom). Carbohydrate utilization by bifidobacteria was examined in de Man Rogosa and Sharpe Medium (MRS) prepared from first principles^18^. Prior to inoculation MRS was supplemented with cysteine-HCl (0.05% w/v) and a particular carbohydrate source (0.5% w/v). The carbohydrates used were lactose and sialic acid (purchased from Sigma), N-Acetyl-D-glucosamine-6-O-sulfate (purchased from Dextra laboratories, Reading, UK), Lacto-*N*-tetraose (purchased from Elicityl, Crolles, France) and Lacto-*N*-neotetraose (obtained from Glycom, Lyngby, Denmark). Bifidobacterial cultures were incubated at 37 °C under anaerobic conditions which were maintained using an anaerobic hood (Davidson and Hardy, Belfast, Ireland). *Escherichia coli* was cultured in Luria Bertani broth (LB)^19^ at 37 °C with agitation. Where appropriate growth media contained tetracycline (Tet; 15 μg ml^−1^), chloramphenicol (Cm; 5 μg ml^−1^ for *E*. *coli* or 2.5 μg ml^−1^ for *B*. *breve*), Spectinomycin (Spec; 100 μg ml^−1^ for *E*. *coli* or *B*. *breve*) or kanamycin (Km; 50 μg ml^−1^ for *E*. *coli*). Recombinant *E*. *coli* cells containing pBS423∆rep were selected on LB agar containing Spec.

**Table 3:** Bacterial Strains and Plasmids used in this study.

Strain or plasmid	Relevant features	Reference or source
**Strains**		
*Escherichia coli* strains		
*E*. *coli* EC101	Cloning host, repA^+^ km^r^	^29^
*E*. *coli* EC101-pNZ-M.BbrII + M.BbrIII	EC101 harbouring pNZ8048 derivative containing *bbrIIM* and *bbrIIIM*, Cm^r^	^25^
*Bifidobacterium* sp. strains		
*B*. *breve* UCC2003	Isolate from nursling stool	^21^
*B*. *breve* UCC2003-nagA1-(I)	UCC2003 pBS423Δrep first crossover integrant via nagA1 deletion fragment I, Spec^r^	This study
*B*. *breve* UCC2003-nagA1-(II)	UCC2003 pBS423Δrep first crossover integrant via nagA1 deletion fragment II, Spec^r^	This study
*B*. *breve* UCC2003-nagA2-(I)	UCC2003 pBS423Δrep first crossover integrant via nagA2 deletion fragment I, Spec^r^	This study
*B*. *breve* UCC2003-nagA1-(I)-pRTB101	*B*. *breve* UCC2003-nagA1-(I) harbouring pRTB101, Spec^r^, Cm^r^	This study
*B*. *breve* UCC2003-nagA1-(II)-pRTB101	*B*. *breve* UCC2003-nagA1-(II) harbouring pRTB101 Spec^r^, Cm^r^	This study
*B*. *breve* UCC2003-nagA2-(I)-pRTB101	*B*. *breve* UCC2003-nagA2-(I) harbouring pRTB101 Spec^r^, Cm^r^	This study
*B*. *breve* UCC2003ΔnagA1	*nag*A1 846 bp inframe deletion mutant of UCC2003	This study
*B*. *breve* UCC2003ΔnagA2	*nag*A2 969 bp inframe deletion mutant of UCC2003	This study
*B*. *breve* UCC2003ΔnagA1ΔnagA2	*nag*A1, *nag*A2 double deletion mutant of UCC2003	This study
UCC2003PK1	*B*. *breve* UCC2003 harbouring pPKCM Cm^r^	^21^
UCC2003ΔnagA1 + 2PK1	*B*. *breve* UCC2003ΔnagA1ΔnagA2 harbouring pPKCM Cm^r^	This study
*B*. *bifidum* ATCC29521(PRL2010)	*B*. *bifidum* type strain	ATCC
*B*. *bifidum* PAM5	*B*. *bifidum* ATCC29521 harbouring pAM5 Tet^r^	This study
Plasmids		
pBS423Δrep	4.4 kb, E. coli- vector, ΔpMB1, ori pTB4 ori repA Spec^r^	^30^
pRTB101	7.3 kbp, E. coli-Bifidobacterium shuttle vector, pMB1 ori pTB4 ori repA	^30^
pPKCM	pCIBA089-pSK-Cm^r^	^31^
pAM5	pBC1-puC19-Tet^r^	^32^

ATCC’ American type culture collection.

### Nucleotide sequence analysis

Sequence data were obtained from the Artemis-mediated^20^ genome annotations of the *B*. *breve* UCC2003^21^. Database searches were performed using non-redundant sequences accessible at the National Centre for Biotechnology Information internet site (http://www.ncbi.nlm.nih.gov) using Blast.

### DNA manipulations

Chromosomal DNA was isolated from *B*. *breve* UCC2003 as previously described^22^. Minipreparation of plasmid DNA from *E*. *coli* or *B*. *breve* was achieved using the Qiaprep spin plasmid miniprep kit (Qiagen GmBH, Hilden, Germany). For *B*. *breve* an initial lysis step was included whereby cells are incubated in lysis buffer containing 30 mg ml^−1^ of lysozyme for 30 minutes at 37 °C. Single stranded oligonucleotide primers used in this study were synthesized by Eurofins (Ebersberg, Germany). Standard PCRs were performed using TaqPCR mastermix (Qiagen), while high fidelity PCR was achieved using Q5 DNA polymerase (New England Biolabs, Ipswich, MA, United States). *B*. *breve* colony PCRs were performed as described previously (O’Connell Motherway *et al*.^17^). PCR fragments were purified using the Roche high pure PCR purification kit (Roche, Hilden, Germany). Electroporation of plasmid DNA into *E*. *coli* was performed as described by Sambrook *et al*.^19^ and into *B*. *breve* UCC2003 as described by Maze *et al*.^23^.

### Construction of *B*. *breve* UCC2003 deletion mutants

Isogenic non-polar deletion mutants of *nag*A1 (Bbr_0846), *nag*A2 (Bbr_1247) or a double deletion strain harbouring deletions in both *nag*A1 and *nag*A2, with 846 bp of the 1245 bp of *nag*A1, or 969 bp of the 1278 bp of *nag*A2 deleted, were created using pBS423Δrep constructs and generated by the splicing by overlap extension (SOEing) PCR procedure^24^. In each case primers SOE AB and SOE CD (Table 4) were used to amplify regions flanking the sequence to be deleted using genomic DNA of *B*. *breve* UCC2003 as template. The resulting products, designated I or II were purified, mixed in a 1:1 ratio and used as template with primers SOE EF. The resulting product was digested with Pst1 and ligated to similarly digested pBS423Δrep prior to transformation into *E*. *coli* EC101 by electroporation. Transformants were selected based on resistance to Km and Spec, and screened by colony PCR using primers pBSF and pBSR to identify clones harbouring the correct insert. The presence of the correct insert in a number of positive clones was confirmed by plasmid isolation and restriction analysis, while the sequence integrity of the cloned DNA fragment and the orientation of the insert in the pBS423Δrep vector was confirmed by sequencing. First crossover insertion mutations were generated essentially as described previously^25^ to produce *B*. *breve* UCC2003 derivatives that were designated UCC2003-nagA1-(I), UCC2003-nagA1-(II), or UCC2003-nagA2-(I), respectively where I or II indicate that the first crossover occurred via fragment I or II (described above). Site-specific recombination in potential spec-resistant mutant isolates was confirmed by colony PCR using primer combinations specFw and specRv to verify Spec^r^-encoding gene integration, and primers nagA1SOE A or nagA2SOE A (positioned upstream of the selected flanking regions of *nagA1* or *nagA2* respectively), each in combination with pBSR or to confirm integration at the correct chromosomal location. To promote pBS423Δrep plasmid excision in UCC2003-nagA1-(I), UCC2003-nagA1-(II), or UCC2003-nagA2-(I) the incompatible plasmid pRTB101-CM was introduced into each strain and transformants were selected on RCA supplemented with CM. CM resistant colonies were subcultured for eight transfers to promote loss of integrated pBS423Δrep. Cells which had excised pBS423Δrep and had either reverted to the wild type genotype, or harboured a *nag*A1 or *nag*A2 deletion, were selected based on Cm^r^ Spec^s^. Screening of Spec^s^ colonies for UCC2003 derivatives harbouring *nag*A1 or *nag*A2 deletion was performed by colony PCR using primer pairs nagA1SOEA and nagA1SOEB or nagA2SOEA and nagA2SOEB, respectively, and sequencing of the PCR products to confirm the inframe deletion. Curing of pRTB101-CM from *B*. *breve* deletion mutant strains was performed by subculturing at 42 °C for 8 transfers followed by plating on RCA and screening for CM sensitive mutant strains by replica plating. Construction of the double deletion strain *B*. *breve* UCC2003ΔnagA1ΔnagA2 was performed sequentially whereby the deletion strain UCC2003ΔnagA2 was constructed first and this strain was used as host for construction of a deletion of *nag*A1.

**Table 4:** Oligonucleotide Primers used in this Study.

Purpose	Primer	Sequence^a^
Construction of UCC2003 nagA1 deletion	nagA1SOE A	catctggtgctgctcgctttcg
	nagA1SOE B	agtgagcagcacgtcggcggccgccacatcgatgccatccg
	nagA1SOE C	cggatggcatcgatgtggcggccgccgacgtgctgctcac
	nagA1SOE D	ctcaaggctgcgatcgacatg
	nagA1SOE E	cgctcactgcagcacccgcacgccacgatcatc
	nagA1SOE F	atctccctgcagctcaaggctgcgatcgacatg
Construction of UCC2003 nagA2 deletion	nagA2SOE A	gcatccgcacgccacgattatc
	nagA2SOE B	caccaaacctgttcgaccgtccaatagccaggagtcaggattcg
	nagA2SOE C	cgaatcctgactcctggctattggacggtcgaacaggtttggtg
	nagA2SOE D	gatctacggcatcaatgagc
	nagA2SOE E	aactgcctgcagcgctacgcatacacccacaag
	nagA2SOE F	atctccctgcagatcattcgttcctgtgcgctttg
pBS423 multiple cloning site primers	pBSF	cgttacgttattagttat
	pBSR	gtaatacgttcgtgtcgcg
Amplification of spectinomycin resistance cassette	specFw	gtcgtcgtatctgaacc
	specRv	gataactacgaactgctaac

^a^Restriction sites incorporated into oligonucleotide primer sequences are indicated in bold.

### Mother-to-pup transmission of *B*. *bifidum* and *B*. *breve*

All experiments using mice were approved by the University College Cork animal ethics committee and all methods were performed in accordance with the relevant guidelines and regulations. Twelve-week-old pregnant female, germ-free C57BL/6 mice were housed in flexible film gnotobiotic isolators under a strict 12 h light cycle. Mice were fed an autoclaved standard polysaccharide-rich mouse chow diet. At approximately 11 days of gestation two groups of mice (n = 7 per group) were inoculated with 1 × 10^9^ cfu of *B*. *bifidum* PAM5 and *B*. *breve* UCC2003PK1, or *B*. *bifidum* PAM5 and *B*. *breve* UCC2003∆nagA1 + 2PK1 in 20 μl of PBS by oral pipetting whereby the inoculums are delivered by positioning a micropipette tip immediately behind the incisors. Five mice were maintained as uninoculated controls to monitor the germ free status of the facility. Fecal pellets were collected weekly to determine the density of bacterial colonisation of each strain based on colony forming units. At approximately 9 days after inoculation the first litter of pups was born and all litters were born within the following 5 days. Half of each dam-reared litter was sacrificed at each of two time points, while the mice were exclusively dam-fed, the first timepoint was at postnatal day 8–11, with the second four days later when mice aged between 12 and 16 days (Table 1). The stomachs of all culled pups were inspected to ensure no chow particles were present. Enumeration of bifidobacterial strains in the caecum and large intestine of each pup was performed using antibiotic selection and plate counting. Enumeration of the bifidobacterial strains in the caecum and large intestine of each mother was also determined at the end of the trial period.

### Statistical analysis

Results are presented as mean +/− SEM. Findings were statistically evaluated using one-way Anova with Dunnett’s post-test. The p value, p < 0.0001, p < 0.001 or p < 0.01 is indicated by three, two or one stars (*), respectively as appropriate. ns indicates non-significant.

### Transcriptional profiling of *B*. *breve* UCC2003 and UCC2003∆nagA1 + 2PK1

Large intestine samples from each group of neonatal pups were snap frozen in liquid nitrogen. After thawing in RNA protect solution each tissue sample was homogenised to separate the luminal contents from the mucosa, and centrifuged (1,200 × g for 10 sec) to remove the tissue fragments. The resulting bacterial supernatants were subjected to bacterial cell lysis and RNA isolation as described previously^21,26^. cDNA from bacterial mRNA was synthesized using the cDNA synthesis and labelling kit DSK-001 (Kreatech, Amsterdam, Netherlands) according to the manufacturer’s instructions and labelled with Cy3 or Cy5 using Cy3-ULS and Cy5-ULS from the cDNA synthesis and labelling kit DSK-001 (Kreatech). DNA-microarrays containing oligonucleotide primers representing each of the 1864 annotated genes on the genome of *B*. *breve* UCC2003 were designed by and obtained from Agilent Technologies (Palo Alto, Ca., USA). Labelled and amplified cDNA was hybridized using the Agilent Gene Expression hybridization kit (part number 5188–5242) as described in the Agilent Two-Color Microarray-Based Gene Expression Analysis (v4.0) manual (publication number G4140–90050). Notably, we do not see cross hybridisation of *B*. *bifidum* cDNA with the *B*. *breve* oligos on these arrays^9^. Following hybridization, microarrays were washed were washed in accordance with Agilent’s standard procedures and scanned using an Agilent DNA microarray scanner (model G2565A). Generated scans were converted to data files with Agilent’s Feature Extraction software (Version 9.5). DNA-microarray data were processed as previously described^27^. Differential expression tests were performed with the Cyber-T implementation of a variant of the *t*-test^28^. A gene was considered differentially expressed when p < 0.001 and an expression ratio of >2 or <0.33 relative to the control. The microarray data have been deposited in NCBI’s Gene Expression Omnibus and are accessible through GEO Series accession number GSE110077.

## Electronic supplementary material

Table S1
