# Supplementary material for: Carbohydrate Syntrophy enhances the establishment of Bifidobacterium breve UCC2003 in the neonatal gut
Source: Sci Rep. 2018 Jul 13;8:10627. doi: 10.1038/s41598-018-29034-0 (PMC6045583; doi:10.1038/s41598-018-29034-0)
Supplement: Supplementary file 1 — Table S1 [file 41598_2018_29034_MOESM1_ESM.docx]

**Carbohydrate Syntrophy enhances the establishment of *Bifidobacterium breve***

**UCC2003 in the neonatal gut**

Mary O’Connell Motherway ^1,2^ , Frances O’Brien^1^_,_ Tara O’Driscoll^1^, Patrick G. Casey^1^, Fergus Shanahan ^1,3^ and Douwe van Sinderen ^1,2^

APC Microbiome Ireland^1^, Schools of Microbiology^2^ and Medicine ^3^ , National University of Ireland, Cork, Western Road, Cork, Ireland.

**Supplementary Table S1**. Genes upregulated in both *B. breve* UCC2003PK1 and *B. breve* UCC2003∆nagA1∆nagA2PK1 during co-colonisation with *B. bifidum* ATCC29521PAM5 in the intestine of dam reared neonatal pups

|  |  | ***B. breve* UCC2003PK1** | | ***B. breve* UCC2003∆nagA1∆nagA2PK1** | |  |
| --- | --- | --- | --- | --- | --- | --- |
| **Name** | **function** | **Fold upregulation** | **pValue** | **Fold upregulation** | **pValue** | |
| **Bbr_0345** | ATP-binding protein of ABC transporter system | 4.66 | 0.0005 | 3.16 | 0.0020 | |
| **Bbr_0620** | DNA-(apurinic or apyrimidinic site) lyase | 15.93 | 0.0006 | 10.00 | 0.0005 | |
| **Bbr_0855** | Hypothetical protein | 5.14 | 0.0002 | 3.76 | 0.0001 | |
| **Bbr_0861** | Conserved hypothetical protein in phosphoglycerate mutase family | 7.80 | 0.0004 | 3.99 | 0.0013 | |
| **Bbr_0875** | Hypothetical membrane spanning protein | 8.33 | 0.0205 | 4.36 | 0.0010 | |
| **Bbr_1071** | Hypothetical membrane spanning protein | 4.40 | 0.0002 | 3.24 | 0.0027 | |
| **Bbr_1097** | hisI Phosphoribosyl-AMP cyclohydrolase | 4.73 | 0.0003 | 3.51 | 0.0041 | |
| **Bbr_1161** | Nucleotide pyrophosphatase | 15.03 | 0.0001 | 9.22 | 0.0000 | |
| **Bbr_1188** | miaA tRNA delta(2)-isopentenylpyrophosphate transferase | 15.63 | 0.0010 | 10.20 | 0.0008 | |
| **Bbr_1277** | Conserved hypothetical membrane spanning protein | 19.48 | 0.0005 | 9.46 | 0.0008 | |
| **Bbr_1286** | acyP Acylphosphatase | 3.72 | 0.0003 | 2.72 | 0.0006 | |
| **Bbr_1514** | Narrowly conserved hypothetical protein | 18.00 | 0.0000 | 7.71 | 0.0002 | |
| **Bbr_1520** | pacB Penicillin acylase | 11.35 | 0.0015 | 5.57 | 0.0022 | |
| **Bbr_1527** | Narrowly conserved hypothetical membrane spanning protein | 12.14 | 0.0001 | 6.66 | 0.0005 | |
| **Bbr_1780** | Glutamyl-tRNA synthetase | 14.60 | 0.0010 | 6.16 | 0.0023 | |
